# Opioid Overdose-Induced Diffusion Restriction in the Bilateral Basal Ganglia Revealed on Brain Imaging

**DOI:** 10.7759/cureus.59649

**Published:** 2024-05-04

**Authors:** Dan Y Draytsel, Sedat Gul, Avishek Sanjel Chhetri, Hesham Masoud

**Affiliations:** 1 Neurology, Upstate University Hospital, Syracuse, USA

**Keywords:** opiod abuse, ischemic stroke workup, basal ganglia, diffusion restriction, opioid overdose

## Abstract

Opioid misuse and addiction have led to an opioid epidemic in the United States, with widespread effects on the healthcare system. Opioid-induced cardiovascular morbidity and mortality effects have been extensively described in past literature; however, neurological effects have been described less frequently.

Here, we describe a case of a female patient who presented to our center after being found unresponsive with magnetic resonance imaging (MRI), revealing bilateral basal ganglia diffuse restriction hyperintensities secondary to a diagnosis of opioid overdose.

Opioid overdose-induced bilateral basal ganglia diffusion restriction has only been described infrequently in the literature. Recognizing the associated imaging findings as a potential consequence of opioid overdose is important to avoid unnecessary workups for ischemic stroke.

## Introduction

Opioid misuse and addiction are a public health crisis resulting in debilitation, deaths, and significant social and economic impacts [1]. Labeled as an opioid epidemic and declared a national public health emergency in 2017 by the US Department of Health and Human Services, it is one of the most severe public health crises in US history [2, 3]. It is a complex, evolving phenomenon that involves neurobiological vulnerabilities and social determinants of health [2]. Nearly 450,000 Americans have died from an opioid-related overdose between 1999 and 2018 [3]. Approximately one in 100 adults in the US has an active opioid-use disorder (OUD) [3]. It is estimated that another 480,000 people could die from fatal opioid overdoses in the next 10 years [3].

Opioid overdose-related outcomes have received extensive research, though mortality and morbidity effects have been described as mainly cardiovascular in nature. The neurological effects have been described less frequently. Here, we describe a case in which a female presented to our center after being found unresponsive, with an MRI revealing bilateral basal ganglia hyperintensities suggesting diffuse restriction secondary to opioid overdose. 

## Case presentation

Our patient is a 56-year-old female with a medical history of metastatic leiomyosarcoma on chemotherapy, OUD, multiple sclerosis, coronary artery disease status post coronary artery bypass grafting (CABG), hypertension, and chronic kidney disease, with chronic opioid therapy for pain control due to her malignancy. She presented to our center after being found unresponsive in her home with a recent history of increased opioid pain medication in the outpatient setting (fentanyl 75 µg/hr and oxycodone immediate release (IR) 15 mg). She endorsed forgetting to remove one of her fentanyl patches, found on her right thigh, and then removed, prompting the diagnosis of an accidental opioid overdose possibly impacted by a recent initiation of gabapentin. 

While in the emergency department (ED), she was found to have agonal breathing and pinpoint pupils. She was given 6 mg of Narcan, with a slight response. She had an episode of vomiting after administration of the Narcan, with continued confusion and an inability to protect her airway. She was intubated. A CT scan of the head (CTH) was negative for any intracranial hemorrhage or injury. The laboratory workup was significant for hyperkalemia (K 6.6), for which she received five units of insulin. She also received 3 L of crystalloid. Acetaminophen, salicylate, and ethyl alcohol (EtOH) levels were negative. She was admitted to the medical intensive care unit (MICU) for ventilator management and was extubated one day later. 

**Figure 1:**
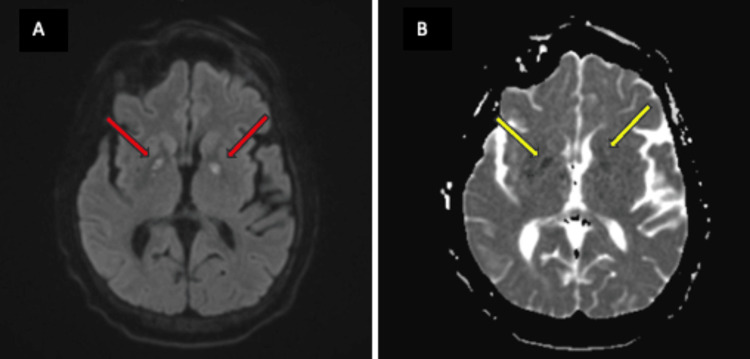
Symmetric bilateral globus pallidus on the brain MRI A: MRI-DWI hyperintensities (red arrows); B: MRI-ADC hypointensities (yellow arrows) MRI: magnetic resonance imaging; DWI: diffuse-weighted imaging; ADC: apparent diffusion coefficient

**Figure 2:**
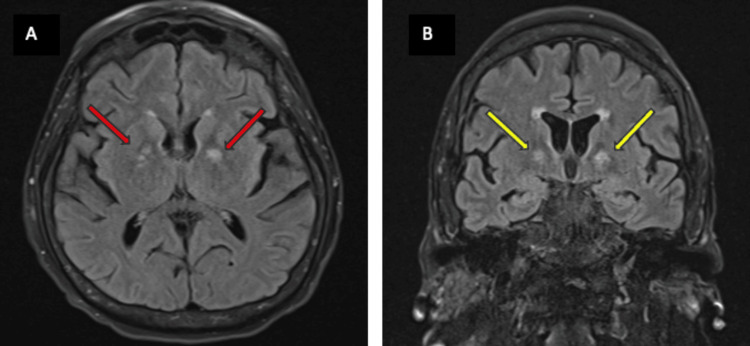
Axial MRI FLAIR (yellow arrows) (A) and coronal MRI FLAIR (yellow arrows) (B) demonstrating corresponding hyperintensities MRI: magnetic resonance imaging; FLAIR: fluid-attenuated inversion recovery

Neurology was consulted for mild confusion after extubation. Her physical exam was only remarkable for her decreased speed of cognitive processing. She underwent a routine electroencephalogram (EEG), which disclosed sharp waves in the bilateral parieto-occipital regions. A video electroencephalogram (VEEG) showed rare, sharp waves from the right frontocentral region without noting a clear seizure pattern. The routine EEG, which was done earlier in her hospitalization, showed some epileptic potential in bilateral hemispheres, but the VEEG, which was done to investigate her status in the long term, showed that epileptic potential decreased over time, likely due to metabolic insult improvement. This also supported the statement that her presentation was not caused by a seizure. A brain MRI with and without contrast was obtained to investigate for metastatic disease, which ultimately revealed the diagnosis of bilateral internal capsule hyperintensities on diffusion-weighted imaging (DWI) and hypointensities in the corresponding apparent diffusion coefficient (ADC) sequence (Figures 1, 2). She did not present with any signs of hypoxic injury or carbon monoxide (CO) poisoning, which are important confounding variables that could cause similar findings on imaging.

Her opioid therapy was reduced to a safer and more reasonable dosage (from 75 µg/hr to 50 µg/hr). She was scheduled to follow up with the pain clinic and was hemodynamically stable at the time of discharge.

## Discussion

Our case is one of the few in the literature to describe opioid overdose-related effects on the neurological system with bilateral basal ganglia diffusion restriction found in this patient. Such knowledge is high yield, considering the high prevalence of current and projected opioid use in the United States and the high toll it takes on our healthcare system. The bilaterality of the hyperintensity is suggestive of a metabolic insult, such as the signature globus pallidus bilateral hyperintensity seen in carbon monoxide poisoning, rather than that of an acute ischemic stroke. It is also very important to note that the diffusion restriction itself does not differentiate between the two etiologies, but the clinical context makes this clearer.

Previously, morbidity and mortality have been described purely from a cardiovascular perspective. Opioids have been found to increase the risk of developing atrial fibrillation (AF), vascular occlusions, and vascular aging (seen via increases in arterial stiffness) while reducing heart rate variability (HRV) [4]. Withdrawal from opioid use, which, when done rapidly, is associated with increases in blood pressure and a decrease in HRV [4].

In a retrospective observational study by Doshi et al., 430,459 patients hospitalized with opioid overdoses were analyzed. They found that 8.6% (36,837) had at least one cardiovascular event, 3.2% (13,979) developed ischemic events (ischemic stroke and myocardial infarction), 0.7% (3,074) developed acute heart failure, and 5.2% (22,444) developed arrhythmias. Opioid overdose patients with new-onset cardiovascular events also had higher odds for in-hospital mortality (odds ratio 4.55, 95% confidence interval: 4.11, 5.04) [5].

Gan et al. performed a nested case-control study and included 16,113 cardiovascular disease (CVD) case subjects and 66,875 control subjects. They found that overdoses that occurred on the index date were strongly associated with CVD, especially arrhythmia, ischemic stroke, hemorrhagic stroke, and myocardial infarction. The CVD risk was decreased by remaining significantly elevated for overdoses that occurred on the previous day and were not observed for overdoses that occurred on each of the previous two to five days [6].

Neurologically, the effects of opioid overdose have been sparingly described. Nelson et al. and Andalibi et al. found that opioid abuse was associated with frequent comorbidities of encephalopathy, neuromuscular disorders, seizures, spine disorders, strokes, central nervous system (CNS) infections, and movement disorders, with opioid use disorder specifically increasing the hazard ratio of stroke mortality without an effect on the disability rate [7, 8]. Birsic et al. described the case of a 37-year-old female with a history of intravenous drug use who presented with extremity weakness and pain and was found to have large endocardial vegetation on both the tricuspid and mitral valves with a patent foramen ovale, prompting embolization of these vegetations into the lungs, liver, and brain [9]. Although her death was a result of endocarditis, her symptoms were similar to those of a stroke [9].

Butt and Nadir described a case of a 30-year-old male who presented with an altered state of consciousness with a Glasgow Coma Scale (GCS) of 3/15 with pinpoint pupils. His blood pressure was 90/60 mmHg, and his respiratory rate was 10 breaths per minute. The CT scan revealed bilateral internal capsule hypolucencies and bilateral frontal lobe infarction. Urinary toxicology screening was positive for extremely high concentrations of opioids and benzodiazepines. He went on to make an uneventful recovery with antidotes and supportive care [10].

Ramierz-Zamora et al. presented the case of a patient who had ingested high doses of oxycodone with an MRI brain within the first 24 hours, revealing symmetric areas of restricted flow in the bilateral pallidal and medial temporal lobes [11].

Specific to the basal ganglia, Hassan et al. described two cases of a 54-year-old male and a 25-year-old male who both presented after being found unconscious at home, with a GCS of 3/15. Both had pinpoint pupils bilaterally. A CT scan revealed the presence of bilateral basal ganglia hypodensities in both patients, in addition to multiple hypodensities scattered in the cerebral hemispheres of one patient. Toxicology screenings were positive for opioids. One patient recovered completely, while the other remained in a vegetative state [12].

Dinicu et al. presented the case of a 16-year-old male who was found unresponsive after ingesting fentanyl-laced oxycodone pills. An MRI revealed distinct areas of restricted diffusion in the bilateral basal ganglia concerning oxidative stress-mediated neuronal loss, in addition to diffuse T2 hyperintensities in the corpus callosum and bilateral frontal, parietal, and cerebellum. His neurological exam improved with supportive treatment [8].

Table 1 compares the age, neurologic symptoms, imaging findings, and clinical outcomes in opioid-overdose case reports.

**Table 1:** Comparison of age, neurologic symptoms, imaging findings, and clinical outcomes in opioid-overdose case reports GCS: Glasgow coma scale; CT: computed tomography; MRI: magnetic resonance imaging; ICU: intensive care unit; IV: intravenous; MICU: medical intensive care unit; AMA: against medical advice

	Age (years)	Sex	Neurologic symptoms	Imaging findings	Clinical outcome
Butt and Nadir [8]	30	Male	Altered state of consciousness, pinpoint pupils, absent doll’s eye movements, upgoing R Babinski sign, GCS 3/15	CT: bilateral internal capsule hypolucencies and bilateral frontal lobe infarction	Uneventful recovery with antidotes and supportive care
Ramirez-Zamora et al. [9]	No mention	No mention	Altered state of consciousness, impaired attention, short-term memory impairment, impaired semantic memory, and reduced spontaneity	MRI: symmetric, areas of restricted diffusion in bilateral globi pallidi interna and both hippocampi	No mention
Hassan et al. (Case1) [10]	54	Male	Altered state of consciousness, unresponsive, bilateral nonreactive pinpoint pupils, GCS 3/15	CT: bilateral hypodensities in the globi pallidi and genus of the internal capsule	Transferred to the ICU after unsuccessful revival with 10.4 mg of IV naloxone, continued supportive care with a regain of consciousness and clinical improvement. Left AMA.
Hassan et al. (Case 2) [10]	25	Male	Altered state of consciousness, unresponsive, bilateral nonreactive pinpoint pupils, hypotonia and hyporeflexia bilaterally in upper and lower extremities, GCS 3/15, spinal myoclonus secondary to brain hypoxia	CT: multiple areas of subcortical and periventricular hypodensities scattered within the bilateral cerebral and cerebellar hemispheres, including the globi pallidi	Intubated with transfer to MICU, GCS improved to 6/15 with 2 mg of naloxone. Went without improvement for two weeks with a tracheostomy and a percutaneous endoscopic gastrostomy tube. Discharged into a rehabilitation center in a vegetative state.
Dinicu et al. [11]	16	Male	Altered state of consciousness, unresponsive, minimum anti-gravity spontaneous movement in all extremities, hyperreflexia with bilateral upgoing Babinski sign	CT: bilateral hypodensities in caudate nuclei; MRI: diffuse T2 hyperintensities in the corpus callosum and bilateral frontal, parietal, and cerebellum; distinct areas of restricted diffusion in the bilateral basal ganglia	Supportive treatment with improvement in the neurological exam and return to baseline in orientation and muscular strength, hyperreflexia, and bilateral upgoing Babinski sign remain unchanged.

Cases such as these are rare, and our case report adds to the growing body of literature illustrating bilateral basal ganglia flow restrictions secondary to opioid overdose. These signs on CT imaging in unconscious patients can be used to prompt physicians to suspect OUD in these patients [12].

Given the immense body of literature surrounding the cardiovascular effects of opioid overdose and the threatening nature of the opioid epidemic in years to come, knowledge of this specific imaging finding secondary to opioid overdose is highly warranted.

## Conclusions

Opioid overdose-induced bilateral basal ganglia diffusion restriction has been described infrequently in the literature. Recognizing this as a potential consequence of opioid overdose is important, as it should not be mistaken for an acute ischemic stroke. It is important to note that diffusion restriction itself does not differentiate between the two etiologies, but the clinical context makes this clearer. Given the high prevalence of estimated fatalities from opioid overdoses in the next 10 years, it becomes paramount to understand some of the nuanced signs and symptoms these overdoses can present with. Doing so will allow physicians to provide a higher standard of care while minimizing excessive spending in our healthcare system. 
